# Enhancing the performance of neurosurgery medical question-answering systems using a multi-task knowledge graph-augmented answer generation model

**DOI:** 10.3389/fnins.2025.1606038

**Published:** 2025-05-20

**Authors:** Ting Pan, Jiang Shen, Man Xu

**Affiliations:** ^1^College of Management and Economy, Tianjin University, Tianjin, China; ^2^Business School, Nankai University, Tianjin, China

**Keywords:** neurosurgery care, intelligent question and answering system, knowledge graph, multi task learning, medical answer generation

## Abstract

**Objective:**

Neurosurgical intelligent question-answering (Q&A) systems offers a novel paradigm to enhance perceptual intelligence—simulating human-like cognitive processing for contextual understanding and emotion interaction. While retrieval-based models lack perceptual adaptability to rare clinical scenarios, and generative LLMs, despite fluency, fail to ground outputs in domain-specific neurosurgical knowledge or doctor expertise. Hybrid frameworks struggle to emulate clinician perceptual workflows (e.g., contextual prioritization, empathy modulation). These present challenges for further improving the semantic understanding, memory integration, and trustworthiness of intelligent Q&A systems in neurosurgery.

**Approach:**

To address these challenges, we propose a Multi-Task Knowledge Graph-Augmented Answer Generation model (MT-KGAG), designed to enhance perceptual fidelity. It uses a hybrid attention mechanism to introduce neurosurgical knowledge graph and doctor features in the answer generation model to prioritize clinically salient information akin to human perceptual workflows. Simultaneously, the model employs a multi-task learning framework, jointly optimizing answer generation, candidate answer ranking, and doctor recommendation tasks aligning machine outputs with clinician decision-making patterns while embedding safeguards against hallucination or inappropriate emotional mimicry. Experiments utilize real-world data from a Chinese online health platform, validated through perceptual coherence metrics and ethical robustness assessments.

**Results:**

The MT-KGAG model outperformed all baselines. It achieved an Embedding Average of 0.9439, DISTINCT-2 of 0.2681, and a medical entity density of 0.2471. Medical experts rated patient safety at 4.02/5 and health outcomes at 3.89/5. Additionally, it attained MRR scores of 0.6155 for candidate answer ranking and 0.6169 for doctor recommendation, confirming its multi-task synergy.

**Discussion:**

MT-KGAG pioneers perception-aware AI in neurosurgery, where LLMs transcend text generation to simulate clinician-like contextual reasoning and ethical judgment. By fusing LLM’s generative adaptability with domain-specific knowledge graphs, the model navigates complex trade-offs between empathetic interaction and perceptual safety—delivering responses that are both contextually nuanced and ethically constrained. This work highlights the transformative potential of perceptual intelligence in medical AI, enabling systems to “interpret” patient needs, “recall” specialized knowledge, and “prioritize” clinical relevance while mitigating risks of anthropomorphic overreach.

## 1 Introduction

The complexity of postoperative care in neurosurgery has escalated significantly in recent years, driven by global demographic shifts toward aging populations and a rising prevalence of chronic diseases. These factors amplify clinical challenges (Dufour and Rousseau-Ventos, 2020). Compounding these issues, a persistent shortage of specialized neurosurgeons continues to hinder access to timely and adequate care, exacerbating unmet demand in healthcare systems (Gebeyehu et al., 2024). To address this challenge, AI-powered medical question-answering (Q&A) systems are emerging as a potential solution. Within the realm of patient care, individuals can engage with AI-powered medical Q&A systems to acquire comprehensive information pertaining to their condition, available treatment options, and postoperative care (Kuang et al., 2023). At their core is the medical answer generation module (MAG) which parses users’ questions and produces appropriate responses in natural language (Iovine et al., 2020). Notably, the clinical adoption of such systems hinges on their ability to emulate human-like perceptual intelligence: contextual understanding, domain-specific reasoning, trustworthiness and ethically constrained interaction—capabilities that remain underdeveloped in current frameworks (Wang et al., 2023). Thus, improving the performance of MAG has been an active area of research (Yang Z. et al., 2021).

MAG models generally fall into three categories. The first category involves retrieval-based Q&A models (Li and Yao, 2022), which operate by matching user questions against pre-constructed question-answer repositories to deliver controlled and interpretable responses (Yang Z. et al., 2021). While delivering controlled responses from predefined repositories, they lack the perceptual adaptability to handle rare cases or dynamic clinical narratives, reflecting rigid knowledge boundaries (Ni et al., 2022). The second category employs generative models that utilize pre-trained large language models (LLMs) as encoder-decoder frameworks (Li L. et al., 2021). They generate contextually coherent and personalized responses through sequential decoding of dialogue history (Schmidgall et al., 2024; Wang et al., 2024). However, their output quality substantially degrades when operating with insufficient training corpora, particularly manifesting as factual inaccuracies or hallucinated content in low-resource clinical contexts. The third category is hybrid models, which combine retrieval-based and generative models to improve accuracy. Typically, retrieval-based models first retrieve candidate answers, while generative models generate new responses, and a ranking model then selects the final output. Early ranking methods relied on feature engineering (Song et al., 2018), but recent approaches increasingly use deep learning (Glass et al., 2022; Yang et al., 2019), enhancing flexibility and accuracy.

While hybrid models have gained research attention, they exhibit two critical limitations. First, prevailing approaches narrowly focus on textual question-answer matching while neglecting perceptual anchors—structured neurosurgical knowledge graphs and physician expertise profiles—that clinicians inherently rely on to contextualize decisions. This oversight severely restricts models’ ability to emulate human-like domain awareness, leading to degraded accuracy when handling complex queries requiring multisource knowledge integration. Second, traditional frameworks isolate retrieval and generative components, reducing answer selection to a binary choice rather than enabling cognitive synergy. Clinicians, conversely, interleave evidence retrieval (e.g., guidelines) with adaptive reasoning (e.g., patient history) in a fluid perceptual loop. Current models fail to mirror this integration, resulting in fragmented semantic representations that struggle to resolve long-tail queries like rare complication scenarios. These gaps underscore the need for architectures that emulate clinician-like perceptual synthesis—seamlessly fusing domain knowledge, contextual cues, and ethical constraints to navigate neurosurgery’s cognitive complexity (Izhar et al., 2025).

To address these challenges, the study proposes the Multi-Task Knowledge Graph-Augmented Answer Generation model (MT-KGAG), which pioneers perception-aware AI for neurosurgical Q&A. The model introduces two innovations. One, it enhances domain-specific understanding by incorporating neurosurgical knowledge graphs (Varshney et al., 2023; Zhao et al., 2022) and doctor features (Shen et al., 2023) into the model. Specifically, an improved relational graph attention network (RGAT) encodes the knowledge graph and feeds it into the decoder, while a hybrid attention mechanism considers doctor features when encoding questions and candidate answers. The neurosurgical knowledge graph provides rich contextual information about medical entities and their relationships (Zhang et al., 2019), while doctor features help assess the expertise and reliability of different doctors in addressing specific medical questions, thereby akin to clinician perceptual reasoning (Shen et al., 2023).

Two, MT-KGAG adopts a multi-task learning (MTL) framework, jointly training question-answer matching, answer generation, and doctor recommendation tasks. MTL is a machine learning paradigm that enhances generalization and performance by enabling models to share knowledge across related tasks, thereby reducing overfitting and improving real-world effectiveness (Ide and Kawahara, 2021; M. Yang Z. et al., 2021; Zhang and Yang, 2021). In recent years, MTL has been widely applied across fields such as computer vision and natural language processing (Wang et al., 2022; Xu et al., 2021), demonstrating particular potentials in Q&A systems and generative tasks (Li Y. et al., 2021; Liu et al., 2024; Zhao et al., 2023). Under this framework, MT-KGAG can align machine outputs with clinician decision-making patterns, the model ensures responses balance factual precision (retrieval) and contextual adaptability (generation), while embedding safeguards against hallucination or inappropriate emotional mimicry—critical for maintaining ethical robustness in patient interactions.

The performance of MT-KGAG is evaluated through comparative analysis and ablation studies. The comparative analysis involves four baseline models: MKGA-DM-NN (Shen et al., 2023), MedPIR (Zhao et al., 2022), AliMe Chat (Qiu et al., 2017), and HybridNCM (Yang et al., 2019). The first model is a retrieval-based Q&A model, the second is a generative model, while the third and fourth are hybrid models. These baselines provide a strong benchmark for assessing the MT-KGAG model’s effectiveness. To further validate the contributions of different components, three ablation versions are designed for comparison: (i) removing multi-task learning, (ii) removing the medical knowledge graph, and (iii) removing doctor-specific features. By conducting these ablation studies, this chapter analyzes the impact of each component on answer quality and further demonstrates the model’s advantages across Q&A matching, answer generation, and doctor recommendation tasks.

This research holds both theoretical and practical significance. Theoretically, the proposed MT-KGAG model it advances medical answer generation (MAG) systems beyond text generation to simulate holistic perceptual workflows—interpreting patient queries through neurosurgical knowledge graph, recalling analogous clinical cases from doctor expertise profiles, and recommending contextually relevant specialists, thereby mirroring the cognitive synthesis inherent to expert decision-making. Practically, the MT-KGAG model provides a more intelligent and efficient automated medical Q&A system for neurosurgery. By dynamically aligning user questions with domain-specific knowledge graphs and clinician historical data, the system improves precision in answer generation—enabling postoperative patients to access tailored, evidence-based guidance with minimal latency (Yan et al., 2024). Furthermore, its advanced recommendation engine employs context-aware personalization, analyzing individual patient histories and clinical nuances to identify optimal physician matches, thereby fostering more relevant and empathetic doctor-patient dialogues.

## 2 Methodology

### 2.1 Framework

To address the limitations mentioned in the introduction, this section proposes the MT-KGAG model. The model consists of three components: (i) candidate answer retrieval, (ii) encoding, and (iii) multi-task training. The encoding and multi-task training components of the model have two distinctive features.

The first feature of the MT-KGAG model is that it effectively integrates medical knowledge graphs and doctor characteristics into the model to enhance its medical understanding capabilities. Specifically, in the first step, this model employs a BERT encoder to encode the questions, candidate answers, and candidate doctors, transforming textual representations into deep semantic vectors, while using an improved Relational Graph Attention Network (RGAT) encoder to encode the knowledge graph. In the second step, after processing by the encoders, the model’s attention mechanism combines self-attention and cross-attention mechanisms to enable deep interaction between the questions, candidate answers, and candidate doctors. This strengthens the model’s understanding and utilization of medical knowledge and doctor characteristics. Through these two designs, the model can effectively extract key information from the knowledge graph and interact with patient questions and doctor information, enabling model-generated answers to better understand patients’ needs, simulate doctors’ perceptions, and improve patients’ perceived trust.

The second feature of the MT-KGAG model is its adoption of a multi-task training strategy to emulate clinician decision-making, jointly training the tasks of answer generation, candidate answer ranking, and doctor recommendation. Specifically, A GPT-based decoder with a dynamic gating mechanism balances generative creativity and evidence-based retrieval, akin to doctors weighing protocols against patient-specific factors. Concurrently, fully connected layers compute relevance scores for answers and doctors, simulating associative memory retrieval and expertise prioritization. This triadic synergy—grounded in knowledge graphs and physician credibility metrics—ensures outputs adhere to neurosurgical best practices while mitigating hallucination risks, embodying ethical perception through transparent, patient-aligned interactions.

**FIGURE 1:**
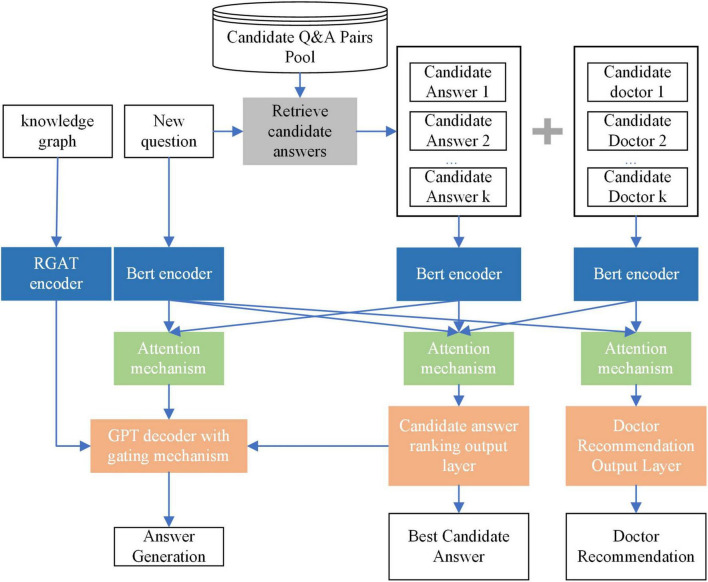
Schematic diagram of MT-KGAG.

By combining these three major components, the MT-KGAG model not only improves the overall performance in question-answer matching and answer generation but also provides more personalized services for OHP, particularly in complex medical scenarios. It simultaneously addresses long-tail problems, doctor recommendations, and accurate answer generation. A schematic diagram of the MT-KGAG model is shown in Figure 1.

### 2.2 Retrieve candidate answer

The retrieval process consists of two sequential stages: disease category classification followed by candidate answer retrieval. In the first stage, both user questions and Q&A pairs from the database are classified into specific disease categories through two complementary methods:

Regular Expression-based Method: This approach employs keyword pattern matching to identify correspondences between question/answer content and named entities in the medical knowledge graph. When matches are detected (e.g., “glioblastoma” or “intracranial aneurysm” in neurosurgical contexts), the system assigns the corresponding disease category. For example, a question containing “persistent headache” would be associated with “brain tumor” through the knowledge graph entity linking “headache” → “brain tumor” in neurosurgical ontology.

Semantic Soft Matching Method: When exact pattern matching fails, we implement semantic similarity computation. Taking a user question *q* as an example, the following is the calculation procedure:

Use word2vec to vectorize the words $w_{i}^{q}$ in *q* and the named entities *v*_*j*_ in the knowledge graph, $e_{i}^{q}=\mathrm{Word2vec}\,\left(w_{i}^{q}\right)$, $e_{j}^{v}=\mathrm{Word2vec}\,\left(v_{j}\right)$;

Calculate the cosine similarity of $e_{i}^{q}$ and $e_{j}^{v}$ by Equation 1 to measure how similar they are:


(1) $$s\,i\,m\,\left(e_{i}^{q},e_{j}^{v}\right)=\frac{e_{i}^{q}\cdot e_{j}^{v}}{\left|e_{i}^{q}\right|\cdot \left|e_{j}^{v}\right|}$$

Select the most matching disease category*C*: Among all the cosine similarities of ${\text{e}}_{i}^{q}$ and ${\text{e}}_{j}^{v}$, select the named entity with the highest similarity, whose disease category is the disease category for question *q*:


(2) $$C=\mathrm{argmax}\,s\,i\,m\,\left(e_{i}^{q},e_{j}^{v}\right)$$

Once *q* is classified into category *C* (as per Equation 2), the system retrieves the top *K* most similar questions from the Q&A database *q*_1_,*q*_2_,*q*_*i*_,*q*_*n*_, all of which belong to *C*. The corresponding answers to these questions serve as candidate responses. The retrieval process involves the following steps:

(1) Vectorized questions, the words of each question in the Q&A database are vectorized by Word2vec, and the vectorized representation of the whole question is obtained by taking the average value. For each question *q_i_*, its vector representation is ***E***_*qi*_.

(2) Calculate the cosine similarity between user question *q* and each question *q_i_*:


(3) $$s\,i\,m\,\left(E_{q},E_{q_{i}}\right)=\frac{E_{q}\cdot E_{q_{i}}}{\left|E_{q}\right|\cdot \left|E_{q_{i}}\right|}$$

(3) The above cosine similarities calculated by Equation 3 are ranked and the answers to the *K* questions with the highest cosine similarity are selected as candidate answers:


(4) $$\begin{array}{c} Top_{k}=X^{1},X^{2},\ldots ,X^{k},\geq \mathrm{sim}((E_{q},E_{q_{1}})=\\ \mathrm{sim}(\left(E_{q},E_{q_{2}}\right)\geq \ldots \geq \mathrm{sim}(\left(E_{q},E_{q_{k}}\right) \end{array}$$

### 2.3 Encoder

In the initial encoding phase, the model encodes the question, candidate answers, doctor text, and knowledge graph. Candidate answers and doctors are obtained from Equation 4. For question, candidate responses and doctor text, the BERT encoder is utilized to encode question, candidate responses and doctor text. Specifically, for question *q*, its embedding is Hq={htqTq}t=1=Bert⁢(Xq), where **X***^q^* represents the word sequence of question *q*, *T^q^* denotes the sequence length, and $h_{t}^{q}$ corresponds to the encoded vector of the *t* -th word in *q*. Similarly, each candidate answer **X***^k^*(1*Sk* = *K*) is encoded as {htkTk}t=1=Bert⁢(Xk), with *T^k^* being the length of **X***^k^* and $h_{t}^{k}$ representing the encoded vector of the *t* -th word in candidate answer **X***^k^*. For doctor text associated with each candidate answer, the encoding is derived from the top 100 high-frequency words in the doctor’s historical answers, yielding {htdkTdk}t=1=Bert⁢(Xdk), where *T^d_k_^* indicates the length of *X^d_k_^* and $h_{t}^{d_{k}}$ stands for the encoded vector of the *t* -th word in doctor *d_k_*. This comprehensive encoding scheme establishes a robust foundation for subsequent processing.

The encoding of the knowledge graph is divided into two stages. In the first stage, medical knowledge subgraph was extracted for each dialogue history $G_{m}^{q}=\left\{V_{m}^{q},E_{m}^{q}\right\}\in G_{m}$. The CMeKG tool is used to extract medical entities from CMeKG, a knowledge graph. It takes every question as an input, and then extracts an approximate match in the set of strings from CmeKG, and finally returns the nodes, which are presented in the medical knowledge graph. Then, these nodes are used as central nodes and the parts with a one-hop relationship are selected to form the subgraph $G_{m}^{q}$.

In the second stage, an improved RGAT was used to encode $G_{m}^{C}$. It can aggregate neighbor node information based on relation-types and highlight key neighbor node information through an improved attention mechanism to fully grasp the internal relationships between nodes. This stage is divided into three steps. In the first step, the input to the encoder is the initial node embedding ${\left({\text{V}}_{m}^{C}\right)}^{0}$, and the embedding method is Bert. The second step calculates the attention weight of each node. The number of RGAT layers is denoted as *L*. Let the input vector of layer *l* be ${\left(V_{m}^{C}\right)}^{l-1}$, then the computation of attention weights in traditional graph attention networks is based only the features of the nodes in Equation 5:


(5) βi⁢jr=LeakyRelu(aT(Wk(vmci)l-1||Wk(vmcj)l-1)

where, $\beta_{i\,j}^{r}$is the attention score, vmci and vmcj denotes the center and neighbor nodes, respectively, ||denotes the connectivity operation, ***a*** is trainable attention weight vector, **W**_*k*_ is weight matrices that are shared linear transformations of the features of each node, and *LeakyRelu*(*ea* is the activation function.

However, this method is unable to take into account the different importance of nodes due to different types of edges between nodes in the attention mechanism, therefore, an improvement is made to the calculation of the attention weights with the following Equations 6, 7:


(6) βi⁢j=LeakyRelu(aT(Wk(vmci)||Wk(vmcj)||Wrei⁢jr)

(7) $$\alpha_{i\,j}={\mathrm{softmax}}_{j}\,\left(\beta_{i\,j}\right)=\frac{e\,x\,p\,\left(\beta_{i\,j}\right)}{\sum_{j^{\prime }\in 𝒩\,\left(i\right)}e\,x\,p\,\left(\beta_{i\,j^{\prime }}\right)}$$

where, *e^r^* is the edge embedding, the embedding method is Bert. ***W***_*r*_ is the trainable matrix. $\alpha_{i\,j}^{r}$ is the attention weight, by normalizing ${\text{β}}_{ij}^{r}$.

In the third step, the node embedding (vmci)l∈(VmC)l is updated. The formula as follows in Equation 8:


(8) (vmci)l=σ⁢(∑j∈𝒩⁢(i)αi⁢j⁢((vmcj)l-1+Wr⁢ei⁢jr))

where σ is the activation function, **W**_*r*_ is the trainable matrix. The edge embedding $e_{i\,j}^{r}$ is set as the initial embedding by Bert of the edge between node *i* and *j*. The node embedding (vmci)l is first calculated by the weighted sum of α_*ij*_ and the node embedding (vmcj)l-1 and $e_{i\,j}^{r}$, and then throughout σ. The final knowledge subgraph is encoded as a concatenation of all node encodings, i.e., $H_{G_{m}^{q}}=c\,o\,n\,c\,a\,t\,\left(V_{m}^{q}\right)$.

### 2.4 Attention mechanism layer

Inspired by Shen et al. (2023), the attention layer is divided into two components: a self-attention mechanism and an interaction-attention mechanism. The self-attention mechanism enables the model to determine the importance of each word within the question, candidate answers, and doctor text. Meanwhile, the interaction-attention mechanism captures the mutual importance among these three elements.

#### 2.4.1 Self-attention mechanism

Specifically, in the self-attention mechanism, a knowledge association matrix ***M*** is introduced into the standard self-attention computation to mine medical relationships in sentences. It is constructed to mine the medical relationships embedded in each sentence. The rows and columns of the matrix are the words in each sentence while the elements represent the medical relationship between the words. The approach is to first match the words with the nodes in the knowledge graph and then determine if there are edges between the nodes. If so, this means there exists a medical relationship between the words. Taking *q* as an example, the expression of the matrix is as follows in Equation 9:


(9) $$m_{i\,j}^{q}=\left\{ \begin{array}{c} e^{r_{i,j}},i\,f\,i⊖j\\ 0,i\,f\,i⊘j \end{array}\right.\;$$

where, *i*⊖*j* denotes *i*,*j* are the nodes that can be retrieved in the knowledge graph and there is an edge between the two. *i*⊘*j* denotes that *i*,*j* are not the nodes that can be retrieved in the knowledge graph or that there is no edge between the two. The self-attention score for question *q* is calculated as follows in Equations 10–14:


(10) $$Q_{s\,e\,l\,f}^{q}=H_{q}\,W_{q}^{q}$$

(11) $$K_{s\,e\,l\,f}^{q}=H_{q}\,W_{k}^{q}$$

(12) $$V_{s\,e\,l\,f}^{q}=H_{q}\,W_{v}^{q}$$

(13) αs⁢e⁢l⁢fqi⁢j=s⁢o⁢f⁢t⁢m⁢a⁢x⁢(t⁢a⁢n⁢h⁢(qs⁢e⁢l⁢fqi⁢||ks⁢e⁢l⁢fqj||⁢Wqr⁢mi⁢jq))

(14) hs⁢e⁢l⁢fq∈Hs⁢e⁢l⁢fq=∑j=1αs⁢e⁢l⁢fqi⁢j⁢vs⁢e⁢l⁢fqj

where, $W_{q}^{q},W_{q}^{k},W_{q}^{v}$ are trainable matrices, with $q_{{\text{self}}_{i}}^{q}\in Q_{\text{self}}^{q}$ and $k_{{\text{self}}_{j}}^{q}\in K_{\text{self}}^{q}$. $\alpha_{s\,e\,l\,f}^{q_{i\,j}}$ represents the self-attention weight score for question *q*, indicating the importance of other words to word *i* within *q*. $m_{i\,j}^{q}$ is an element of the knowledge association matrix **M***^q^* for question *q*. The elements of **M***^q^* reflect the strength and nature of medical relationships between word pairs, rather than syntactic relationships. This matrix is tailored specifically for the medical domain. $H_{s\,e\,l\,f}^{q}$ is the question encoding obtained through the self-attention mechanism.

Similarly, the self-attention scores for candidate answer **X***^k^* and its associated doctor information *d^k^* can be expressed as $\alpha_{s\,e\,l\,f}^{k_{i\,j}}$ and αs⁢e⁢l⁢fdki⁢j, with their respective encodings represented as $H_{s\,e\,l\,f}^{a_{k}}$ and $H_{s\,e\,l\,f}^{d_{k}}$.

#### 2.4.2 Interaction-attention mechanism

First, for question *q*, considering that each candidate answer may contribute differently to the question and that each word within an answer may have varying relevance to the question, we employ a two-layer attention mechanism: global interaction and word-level interaction. Global interaction computes the overall relevance between each candidate answer and the question. Word-level interaction captures the association between individual words within an answer and the question.

The formula for global interaction is as follows in Equations 15–17:


(15) Qqg⁢l⁢o⁢b⁢a⁢l=Hg⁢l⁢o⁢b⁢a⁢lq⁢Wqqg⁢l⁢o⁢b⁢a⁢l

(16) Kakg⁢l⁢o⁢b⁢a⁢l=Hg⁢l⁢o⁢b⁢a⁢lak⁢Wkakg⁢l⁢o⁢b⁢a⁢l

(17) αg⁢l⁢o⁢b⁢a⁢lq⁢ak=s⁢o⁢f⁢t⁢m⁢a⁢x⁢(Qqg⁢l⁢o⁢b⁢a⁢l⁢Kakg⁢l⁢o⁢b⁢a⁢ld)

where, Wqqg⁢l⁢o⁢b⁢a⁢l,Wkakg⁢l⁢o⁢b⁢a⁢l are trainable matrices, while $H_{g\,l\,o\,b\,a\,l}^{q},H_{g\,l\,o\,b\,a\,l}^{a_{k}}$ represent the averaged word encodings of question *q* and candidate answer **X***^k^*, respectively, obtained through the encoder. $\alpha_{g\,l\,o\,b\,a\,l}^{q\,a_{k}}$ denotes the global cross-attention score for candidate answer **X***^k^*.

**FIGURE 2:**
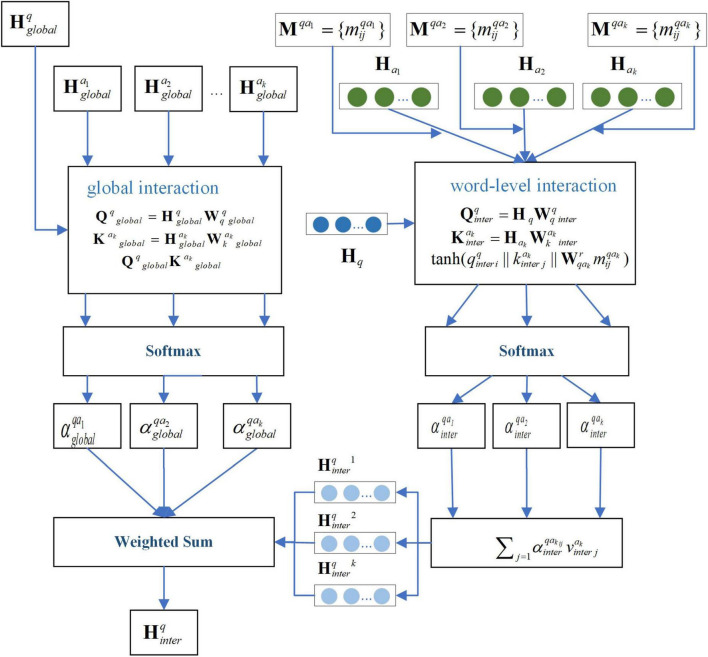
Schematic diagram of the interaction-attention mechanism of the question.

For word-level interaction, the knowledge association matrix **M***^qa_k_^* is incorporated to account for medical relationships when computing attention weights. In this context: The rows of **M***^qa_k_^* correspond to words in question *q*. The columns represent words in candidate answer **X***^k^*. Each element indicates the medical relationship between word pairs. The formula is as follows in Equations 18–22:


(18) Qi⁢n⁢t⁢e⁢rq=Hq⁢Wqqi⁢n⁢t⁢e⁢r

(19) Ki⁢n⁢t⁢e⁢rak=Hak⁢Wkaki⁢n⁢t⁢e⁢r

(20) Vi⁢n⁢t⁢e⁢rak=Hak⁢Wvaki⁢n⁢t⁢e⁢r

(21) αi⁢n⁢t⁢e⁢rq⁢aki⁢j=s⁢o⁢f⁢t⁢m⁢a⁢x⁢(t⁢a⁢n⁢h⁢(qi⁢n⁢t⁢e⁢rqi⁢||ki⁢n⁢t⁢e⁢rakj||⁢Wq⁢akr⁢mi⁢jq⁢ak))

(22) hi⁢n⁢t⁢e⁢rqk∈Hi⁢n⁢t⁢e⁢rqk=∑j=1αi⁢n⁢t⁢e⁢rq⁢aki⁢j⁢vi⁢n⁢t⁢e⁢rakj

where, Wqqi⁢n⁢t⁢e⁢r,Wkaki⁢n⁢t⁢e⁢r are trainable matrices, αi⁢n⁢t⁢e⁢rq⁢aki⁢j denotes the word-level cross-attention score for candidate answer **X***^k^*. Hi⁢n⁢t⁢e⁢rqk represents the encoded question features after word-level cross-attention processing. By combining with the global cross-attention mechanism, the final encoded representation of question *q* through the cross-attention mechanism can be expressed as: hi⁢n⁢t⁢e⁢rq∈Hi⁢n⁢t⁢e⁢rq=∑k=1αg⁢l⁢o⁢b⁢a⁢lq⁢ak⁢hi⁢n⁢t⁢e⁢rqk. Figure 2 illustrates the schematic diagram of the interaction-attention mechanism for the question.

Second, for candidate answer **X***^k^*, two types of interactions are considered. One type is Answer-Question Interaction. It captures the relevance between the answer and the original question. The other type is Answer-Doctor Interaction. It incorporates the influence of the doctor’s expertise into the answer representation.

Taking the Answer-Question Interaction as an example, the interaction attention is calculated as follows in Equations 23–26:


(23) Qi⁢n⁢t⁢e⁢rak=Hak⁢Wqaki⁢n⁢t⁢e⁢r

(24) Ki⁢n⁢t⁢e⁢rq=Hq⁢Wkqi⁢n⁢t⁢e⁢r

(25) Vi⁢n⁢t⁢e⁢rq=Hq⁢Wvqi⁢n⁢t⁢e⁢r

(26) $$\alpha^{a_{k}q_{ij}}{}_{inter}\;=\;softmax\left(tanh\left(q_{inter_{i}}^{a_{k}}||k_{inter_{j}}^{q}||W_{a_{k}q}^{r}m_{ij}^{a_{k}q}\right)\right)$$

where, $m_{ij}^{a_{k}q}$ denotes elements of the knowledge relevance matrix between candidate answer **X***^k^* and question *q*.

Similarly, the interaction attention for Answer-Doctor Interaction is denoted as αi⁢n⁢t⁢e⁢rak⁢dki⁢j. The final encoded representation of candidate answer **X***^k^* after cross-attention is: hi⁢n⁢t⁢e⁢rak∈Hi⁢n⁢t⁢e⁢rak=L⁢i⁢n⁢e⁢a⁢r⁢([∑j=1αi⁢n⁢t⁢e⁢rak⁢qi⁢j⁢vi⁢n⁢t⁢e⁢rq;∑j=1αi⁢n⁢t⁢e⁢rak⁢dki⁢j⁢vi⁢n⁢t⁢e⁢rdkj]). This operation concatenates the outputs of both attention mechanisms and reduces dimensionality via a linear layer to produce a unified fused representation.

Third, for doctor *d^k^*, each doctor has their own specialization, and doctors tend to answer questions within their expertise. Thus, the interaction attention mechanism must account for the role of the question in the doctor’s encoding. The interaction attention is calculated by Equations 28–30:


(27) Qi⁢n⁢t⁢e⁢rdk=Hdk⁢Wqdki⁢n⁢t⁢e⁢r

(28) Ki⁢n⁢t⁢e⁢rq=Hq⁢Wkqi⁢n⁢t⁢e⁢r

(29) Vi⁢n⁢t⁢e⁢rq=Hq⁢Wvqi⁢n⁢t⁢e⁢r

(30) $$\alpha_{i\,n\,t\,e\,r}^{d_{k}\,q_{i\,j}}=s\,o\,f\,t\,m\,a\,x\,\left(t\,a\,n\,h\,\left(q_{i}^{d_{k}}\,||k_{j}^{q}||\,W_{d_{k}\,q}^{r}\,m_{i\,j}^{d_{k}\,q}\right)\right)$$

where, $m_{i\,j}^{d_{k}\,q}$ represents elements of the knowledge association matrix between candidate doctor *d^k^* and question *q*. The encoded representation of candidate doctor *d^k^* after cross-attention is: $h_{i\,n\,t\,e\,r}^{d_{k}}\in H_{i\,n\,t\,e\,r}^{d_{k}}=\sum_{j=1}\alpha_{i\,n\,t\,e\,r}^{d_{k}\,q_{i\,j}}\,v_{j}^{q}$.

#### 2.4.3 Fusion of self-attention and interaction-attention mechanisms

The encodings obtained from the self-attention mechanism and interaction-attention mechanism are fused to produce a combined representation. Taking question *q* as an example, the fusion is calculated by Equation 31:


(31) $$H_{f\,u\,s\,i\,o\,n}^{q}=g⊙H_{s\,e\,l\,f}^{q}+\left(1-g\right)⊙H_{i\,n\,t\,e\,r}^{q}$$

where, gating coefficient *g*is learned through a gating network, *g* controls the weight of the self-attention encoding, while 1 – *g*controls the weight of the cross-attention encoding. The coefficient *g* is calculated using a two-layer MLP by Equations 32, 33:


(32) $$\text{z}=\left[H_{s\,e\,l\,f}^{q};H_{i\,n\,t\,e\,r}^{q}\right]$$

(33) $$g=\sigma \,\left(W_{2}\,R\,e\,L\,U\,\left(W_{1}\,\text{z}+{\text{b}}_{1}\right)+{\text{b}}_{2}\right)$$

where, **z** ∈ ℝ^2*d*^ represents the concatenated vector of self-attention and cross-attention encodings, with *d* being the encoding dimension. The parameters **W**_1_ and **b**_1_ correspond to the first layer’s weight matrix and bias, while **W**_2_ and **b**_2_ belong to the second layer. The *ReLU* activation function introduces nonlinearity, and the sigmoid function σ scales the output to the range [0,1] to produce the gating coefficient *g*. Similarly, the fused encodings for candidate answer **X***^k^* and doctor *d^k^* are denoted as $H_{f\,u\,s\,i\,o\,n}^{a_{k}}$ and $H_{f\,u\,s\,i\,o\,n}^{d_{k}}$, respectively.

### 2.5 Output layer

In the design of the output layer, the main objective is to combine the ability of the model to generate answers with the mechanism of candidate answer selection, so as to optimize the answer generation process and the results of the doctor’s recommendation. Therefore, the output layer has a total of three parts. The first part is the decoding layer, the second part is the candidate answer sorting output layer, and the third part is the doctor selection output layer. Detailed descriptions and formulas for each part are given below.

The first part is the decoding layer, which employs GPT as the decoder and incorporates a dynamic balancing mechanism to flexibly choose between generating new words and retrieving words from the candidate answer. The process involves two operational modes and a gating mechanism to dynamically harmonize generation and retrieval. The first mode is the generation mode. In this mode, the decoder generates words autonomously based on the encoded question semantics and knowledge graph context. The decoder generates each word *y_t_*, with the vocabulary generation distribution defined as pg⁢e⁢n⁢(yt)=s⁢o⁢f⁢t⁢m⁢a⁢x⁢(G⁢P⁢T⁢(st-1,Hf⁢u⁢s⁢i⁢o⁢nq,vmqtt=1Tm⁢q)). The second mode is copy mode, which involves copying words from the candidate answer, where the probability of generating a word ww is given by pc⁢o⁢p⁢y⁢(w)=s⁢o⁢f⁢t⁢m⁢a⁢x⁢(stT⁢Hf⁢u⁢s⁢i⁢o⁢nakm⁢a⁢x). Here, s⁢o⁢f⁢t⁢m⁢a⁢x⁢(stT⁢Hf⁢u⁢s⁢i⁢o⁢nak) measures the similarity between the decoder’s hidden state and the candidate answer **X***^k^*. The indicator function ∃(*w* ∈ **X***^a_k_max__^*) determines whether the word *w* exists in the candidate answer **X***^k^*. A trainable gate dynamically determines the trade-off between generation and copying. The specific formula is as follows in Equations 34, 35:


(34) $$g_{g\,e\,n}=\sigma (W_{g\,a\,t\,e}\left[H_{f\,u\,s\,i\,o\,n}^{q};H_{f\,u\,s\,i\,o\,n}^{a_{k_{m\,a\,x}}}\right]$$

(35) $$p_{f\,i\,n\,a\,l}\,\left(y_{t}\right)=g_{g\,e\,n}\times p_{g\,e\,n}\,\left(y_{t}\right)+\left(1-g_{g\,e\,n}\right)\times p_{c\,o\,p\,y}\,\left(y_{t}\right)$$

where, **W**_*gate*_ and **b** are trainable parameters. $H_{f\,u\,s\,i\,o\,n}^{a_{k_{m\,a\,x}}}$ is the encoding of the best answer computed by the candidate answer ranking layer.

The second part is the Candidate Answer Ranking Layer, which computes a ranking score for each candidate answer based on its relationship with the input question. The question *q* and each candidate answer **X***^k^* are fed into a fully connected layer to obtain their combined representation, from which the ranking score is derived. The formula is as follows in Equation 36:


(36) $$f_{a_{k}}=W_{r\,a\,n\,k}^{a_{k}}•\left[H_{f\,u\,s\,i\,o\,n}^{q};H_{f\,u\,s\,i\,o\,n}^{a_{k}}\right]+b_{r\,a\,n\,k}^{a_{k}}$$

where, $\left[H_{f\,u\,s\,i\,o\,n}^{q};H_{f\,u\,s\,i\,o\,n}^{a_{k}}\right]$ denotes the concatenation of the question and candidate answer encodings, and **W**_*rank*_ represents trainable parameters.

The ranking probability for each candidate answer is calculated by Equation 37:


(37) $$p_{r\,a\,n\,k}\,\left(a_{k}\right)=\frac{e\,x\,p\,\left(f_{a_{k}}\right)}{\sum_{k=1}^{K}e\,x\,p\,\left(f_{a_{k}}\right)}\;$$

where, *p*_*rank*_(*a*_*k*_) indicates the probability that the candidate answer kk is ranked as the best answer.

The third component is the Physician Selection Layer, which integrates the question *q*, candidate answer **X***^k^*, and doctor *d^k^* through a fully connected layer to generate their combined representation. This representation is then used to compute the ranking score for physician selection. The formula is defined as follows in Equation 38:


(38) $$f_{d_{k}}=W_{r\,a\,n\,k}^{d_{k}}•\left[H_{f\,u\,s\,i\,o\,n}^{q};H_{f\,u\,s\,i\,o\,n}^{a_{k}};H_{f\,u\,s\,i\,o\,n}^{d_{k}}\right]+b_{r\,a\,n\,k}^{d_{k}}$$

where, $\left[H_{f\,u\,s\,i\,o\,n}^{q};H_{f\,u\,s\,i\,o\,n}^{a_{k}};H_{f\,u\,s\,i\,o\,n}^{d_{k}}\right]$ denotes the concatenation of the question, candidate answer, and corresponding doctor encodings, while $W_{r\,a\,n\,k}^{d_{k}}$ represents trainable parameters.

The selection probability for each physician is calculated by Equation 39:


(39) $$p_{r\,a\,n\,k}\,\left(d_{k}\right)=\frac{e\,x\,p\,\left(f_{d_{k}}\right)}{\sum_{k=1}^{K}e\,x\,p\,\left(f_{d_{k}}\right)}$$

where, *p*_*rank*_(*a*_*k*_) indicates the probability that doctor *d^k^* is selected as the recommended doctor.

### 2.6 Loss function

In the MT-KGAG model, since it involves three tasks (answer generation, candidate answer ranking, and doctor selection), it is necessary to define an independent loss function for each task and combine these loss functions with weighted summation to form the final total loss for joint training. This multi-task learning approach enables the simultaneous optimization of the objectives of all three tasks. The loss function for the answer generation task typically employs cross-entropy loss to measure the discrepancy between the model-generated answers and the ground truth answers. The loss function for the answer generation task can be defined by Equation 40:


(40) $${ℒ}_{g\,e\,n}=-\frac{1}{N}\,\sum_{n=1}^{N}l\,o\,g\,p_{f\,i\,n\,a\,l}\,\left(y_{t}\right)$$

where, *N* is the sample size of the training dataset, and ℒ_*gen*_ represents the cross-entropy between the predicted answer tokens and the gold answer tokens. Similarly, the loss function for the candidate answer ranking task is defined by Equation 41:


(41) $${ℒ}_{r\,a\,n\,k}=-\frac{1}{N}\,\sum_{n=1}^{N}l\,o\,g\,p_{r\,a\,n\,k}\,\left(a_{k}\right)$$

The loss function for the doctor selection tash is defined by Equation 42:


(42) $${ℒ}_{d\,o\,c}=-\frac{1}{N}\,\sum_{n=1}^{N}l\,o\,g\,p_{r\,a\,n\,k}\,\left(d_{k}\right)$$

Finally, the total loss of the MT-KGAG model is a weighted combination of ℒ_*gen*_, ℒ_*rank*_, and ℒ_*doc*_, defined as follows in Equation 43:


(43) $$ℒ=\frac{1}{2\,\alpha^{2}}\,{ℒ}_{g\,e\,n}+\frac{1}{2\,\beta^{2}}\,{ℒ}_{r\,a\,n\,k}+\frac{1}{2\,\gamma^{2}}\,L_{d\,o\,c}$$

where, α,β,γ are trainable parameters, which can be automatically adjusted during training using the method proposed by Liebel and Körner (2019).

## 3 Experiment

### 3.1 Datasets and knowledge graph

The study employed the dataset from Shen et al. (2023). This dataset was derived from the “Questions and Answers” section of an online health platform.^1^ This platform encompasses a total of 13 primary departments and 46 secondary departments, having accumulated approximately 1.16 million Q&A data. Each response offers insight into the physician’s expertise. By clicking on the doctor’s avatar, users can access the doctor’s homepage and view the doctor’s detailed information, including 100 pairs of recently answered Q&As. The comprehensive collection of Q&A records on the platform, including those specific to neurosurgery, is of paramount importance. This comprehensive data collection process establishes the foundation for the existing Q&A repository. A total of 26,625 Q&A records were collected, involving 3,840 unique user questions. For model development, 60% of the data was used for training, 20% for validation, and the remaining 20% for testing. Based on parameter settings, the number of retrieved candidate answers was set to *K* = 4, meaning each user question is associated with 4 candidate answers and 4 candidate doctors. Table 1 shows the statistical description of the dataset.

**TABLE 1:** The statistic of datasets.

Datasets \ Attribute	Number of user questions	Number of candidate answers	Average length of questions	Average length of answers
Training dataset	2,304	4	32	66
Development dataset	768	4	38	68
Test dataset	768	4	44	69
Total	3,840	15,360	36	68

**FIGURE 3:**
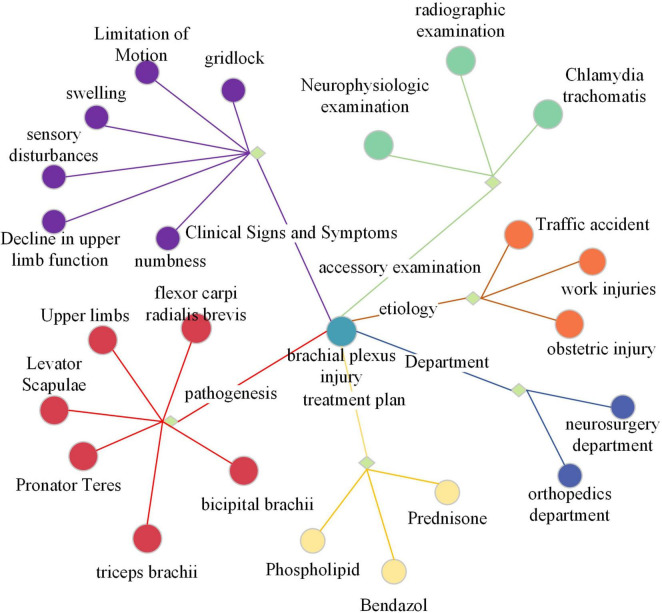
Illustration of the knowledge graph.

The knowledge graph selected is the neurosurgery component of CMeKG. The CMeKG framework facilitates the identification of medical relationships between words and statements within the historical context of medical discourse. This enables the construction of a knowledge association matrix and a knowledge subgraph of the conversation history. Figure 3 offers a partial illustration of the KG. Each node in the graph represents a named entity, with edges denoting the relationships between these entities. The relationships can be broadly classified into nine categories, including UMLS, ICD-10, clinical symptoms and features, site of onset, examination, English name, department, cause of disease, and treatment options.

### 3.2 Baselines

In order to systematically evaluate the proposed model, several representative models from existing studies were selected as baselines. The first model is MKGA-DM-NN (Shen et al., 2023), which can be regarded as the current state-of-the-art Q&A matching model. For the purposes of this study, the model framework was adopted, with modifications to the Q&A matching task, which was altered into an answer ranking task. The model’s first-ranked answer was utilized as the generated response for the metrics calculation. The second model is MedPIR (Zhao et al., 2022), which can represent the current state-of-the-art answer generation model. For this model no calculations are made for candidate answers and doctor choices. The third model is AliMe Chat (Qiu et al., 2017), an open-domain chatbot engine that integrates the joint results of Information Retrieval (IR) and Sequence-to-Sequence (Seq2Seq) based generative models. AliMe Chat employs a Seq2Seq-based fine-grained reordering model to optimize the joint results. The fourth model is HybridNCM (Yang et al., 2019). This model is a hybrid neural dialogue model with response retrieval and generation capabilities. It uses a remote supervision approach to automatically infer the labels of retrieved/generated candidate responses. A rigorous comparison of these four models with the MT-KGAG model can provide a nuanced understanding of their relative strengths and weaknesses.

### 3.3 Evaluation dimension and metrics

In order to assess the performance of each method, the evaluation metrics selected in this chapter focus on eight key dimensions. The first four of these dimensions, accuracy, diversity, knowledge utilization, and fluency, were assessed from a language perspective. Two of the dimensions-patient safety and health outcomes-are evaluated from a healthcare perspective. The last two dimensions-Candidate Answer Ranking Ability and Physician Selection Ability-were assessed from two ancillary task perspectives. The first three dimensions use the embedding mean, DISTINCT, and healthcare named entity density, respectively, which are metrics typically applied to generative models. Fluency, patient safety, and health outcomes are closely related to user experience and cannot be accurately measured by fixed computational metrics, and are therefore evaluated by manual assessment. Candidate answer ranking capability and doctor selection capability are measured utilizing *MRR*.

Specifically, Embedding Average measures the cosine similarity between the text vectors of the answer generation and the text vectors of the real answers, and DISTINCT quantifies the number of unique n-grams in the answer generation. Medical Named Entity Density calculates the ratio of the number of medical named entities to the total number of words. Fluency evaluates the smoothness of answer generation. Patient safety (Denecke and Warren, 2020) examines whether the answer generation causes any harm or risk to the patient. Health outcomes focus on the appropriateness and validity of the answers for the patient. Evaluation of these metrics was independently scored on a scale from 1 (poor) to 5 (excellent) by five evaluators with graduate medical degrees and experience in text mining annotation tasks. The final score was averaged across them. *MRR* is concerned with where the first relevant element appears in the sorted result. If the first relevant element appears in a higher position, the value will be larger. This is calculated by taking the smallest value of the relevant element in the inverse position in the sorted list, and then averaging these values.

### 3.4 Implementation details

In the Retrieve Candidate Answers step, _*K=4*_, i.e., selecting 4 candidate answers for each question. It can preserve the model performance while keeping the model complexity within acceptable limits. All experiments are implemented by Pytorch. For the knowledge subgraph encoder, the node embedding size and speaker embedding size are both 768, and the graph is encoded using 2-layer KRGAT. The learning rate is initialized to 10^−5^. Adam optimizer is used and the learning rate is warmed up in the first 3,000 steps and the learning rate decays linearly. The model generates the response through a beam sampling algorithm where the beam size and top-k are set to 5 and 64, respectively. Other generation hyperparameters are kept at default settings. NVIDIA A100 is used as the computational infrastructure in this chapter. In the future, when our GPUs are equipped with greater memory capacities, such as 50GB, it will become feasible to conduct large-scale model experiments with more than 14B parameters.

## 4 Results and discussion

### 4.1 Comparative analyses

From Table 2, it can be observed that the proposed MT-KGAG model outperforms other baseline models across multiple evaluation metrics. From a linguistic perspective, it achieves the highest scores in accuracy, diversity, and knowledge utilization. This indicates that: First, in terms of answer correctness or relevance, MT-KGAG generates more reliable results by effectively integrating knowledge learned from both retrieval and generative models. Second, MT-KGAG better avoids repetitive or formulaic expressions and leverages candidate answers to provide richer and more flexible phrasing. Third, it successfully incorporates external medical knowledge into the answer generation process, thereby enhancing the professionalism and specificity of responses, which significantly improves knowledge utilization. In terms of fluency, MT-KGAG ranks second, trailing only slightly behind MKGA-DM-NN. This suggests that the model not only ensures the correctness of answers but also produces syntactically coherent and natural language expressions.

**TABLE 2:** Performance for all the models.

Model \ Evaluation	Linguistic perspective				Healthcare perspective		Auxiliary task perspective	
	**Accuracy (embedding average)**	**Diversity (distinct)**	**Knowledge utilization (medical entity density)**	**Fluency**	**Patient safety**	**Health outcome**	**Candidate answer MRR**	**Docter selection MRR**
MKGA-DM-NN	0.8204	0.0957	0.2158	**4.56**	3.28	3.56	0.5441	−
MeREAN	0.7825	0.2245	0.2069	3.86	3.72	3.63	−	−
AliMe Chat	0.8070	0.1783	0.1772	3.95	3.81	3.72	0.4834	0.4512
HybridNCM	0.8351	0.1457	0.1864	2.79	3.05	2.66	0.5017	0.5074
**MT-KGAG**	**0.9439**	**0.2861**	**0.2471**	**4.05**	**4.02**	**3.89**	**0.6155**	**0.6169**

Bold values indicate the best performance for each metric (column-wise comparison).

In comparison, MKGA-DM-NN, while achieving relatively high accuracy, exhibits lower diversity, indicating that its responses are constrained by the answer repository, limiting richness and flexibility. MeREAN performs well in diversity but suffers from lower accuracy, reflecting shortcomings in generation quality. HybridNCM approaches MT-KGAG in accuracy but falls short in diversity and knowledge utilization, suggesting its inability to leverage knowledge graphs to enhance the professionalism of generated answers.

From a healthcare perspective, MT-KGAG demonstrates the best performance in patient safety and health outcomes. This confirms its ability to provide more authoritative and rigorous responses to medical queries, delivering accurate and useful health advice or information. This ethical perceptual rigor stems from its physician feature integration, which filters answers based on specialist credibility, akin to clinicians vetting peer recommendations. MKGA-DM-NN performs poorly in patient safety and health outcomes, indicating that relying solely on candidate answer ranking is insufficient for offering precise medical recommendations and may produce seemingly professional but clinically irrelevant answers. HybridNCM scores significantly lower in healthcare-related metrics, likely due to inadequate coverage of specialized knowledge in its hybrid retrieval-generation approach.

From an auxiliary task perspective, MT-KGAG far surpasses baseline models in both candidate answer MRR and doctor selection MRR, demonstrating that its multi-task design effectively improves the ranking quality of candidate answers and doctor recommendations. AliMe Chat shows moderate performance in these auxiliary tasks but remains limited in professional domains due to its generative nature. HybridNCM performs moderately in auxiliary tasks but still lags behind MT-KGAG overall.

In summary, MKGA-DM-NN is primarily a QA matching-based ranking model that directly outputs the top-ranked answer, relying heavily on similarity modeling while neglecting diversity and flexible knowledge application. MeREAN represents a more advanced generative dialogue model but struggles with fluency and accuracy. AliMe Chat and HybridNCM combine retrieval and generation capabilities but still face limitations in medical knowledge utilization and diversity modeling. In contrast, MT-KGAG, through more effective knowledge graph integration and multi-task collaboration, ensures high answer accuracy and health outcomes while also improving diversity, fluency, and adaptability to medical scenarios. Additionally, it provides a basis for doctor recommendations, making it suitable for future expansion into medical Q&A and recommendation systems.

In conclusion, by embedding perceptual intelligence into every layer—from knowledge fusion to multi-task optimization—MT-KGAG sets a new standard for medical AI, where technical excellence aligns with clinician-like reasoning and ethical accountability. Its performance validates a paradigm shift: systems that “think” like physicians, not just “answer” like databases, are pivotal to scaling trustworthy neurosurgical care globally.

### 4.2 Ablation studies

To evaluate the relative contributions of each component in MT-KGAG to task performance, we conducted an ablation study using the dataset. Specifically, we independently removed multi-task learning, the medical knowledge graph, and doctor features, then compared the performance of each modified model with that of the complete MT-KGAG model. Table 3 presents the results of the ablation study.

**TABLE 3:** Performance of ablation study.

Model \ Evaluation	Linguistic perspective				Healthcare perspective		Auxiliary task perspective	
	**Accuracy (embedding average)**	**Diversity (Distinct)**	**Knowledge utilization (medical entity density)**	**Fluency**	**Patient safety**	**Health outcome**	**Candidate answer MRR**	**Docter selection MRR**
**MT-KGAG**	**0.9439**	0.2861	**0.2471**	**4.05**	**4.02**	**3.89**	**0.6155**	**0.6169**
w/o multi task learning	0.9300	**0.3201**	0.1929	4.00	3.52	3.46	−	−
w/o medical knowledge graph	0.6354	0.1083	0.1047	3.26	3.01	3.13	0.6120	0.6098

Bold values indicate the best performance for each metric (column-wise comparison).

Removing multi-task learning led to declines in accuracy, knowledge utilization, fluency, patient safety, and health outcomes for the generation task. This indicates that multi-task learning plays a crucial role in enhancing the model’s overall performance. Specifically, the candidate answer ranking task directly improves the accuracy and practicality of generated responses by optimizing the relevance and quality of candidate answers. The candidate doctor ranking task enhances the model’s adaptability to personalized medical scenarios by incorporating doctor features.

Multi-task learning strengthens the model’s ability to handle complex medical scenarios by simultaneously optimizing both candidate doctor ranking and candidate answer ranking tasks. Without multi-task learning, the model loses its joint optimization capability for these tasks, resulting in an inability to fully leverage doctor features and candidate answer relevance during response generation. This ultimately reduces the accuracy and professionalism of the responses. Additionally, multi-task learning improves the model’s ability to integrate medical knowledge through shared representations and parameter optimization. Its removal leads to decreased knowledge utilization, further degrading response quality.

Removing the medical knowledge graph caused significant declines in the generation task’s accuracy, diversity, knowledge utilization, fluency, patient safety, and health outcomes. This demonstrates the critical importance of the medical knowledge graph. Without it, the model fails to effectively recognize and represent medical named entities and their relationships, impairing its ability to generate professional medical knowledge. Furthermore, performance on both candidate answer ranking and doctor selection tasks also deteriorates.

The medical knowledge graph provides rich medical background knowledge for candidate answer ranking, enabling the model to more accurately assess answer relevance and quality. Its removal deprives the model of medical knowledge dependencies, reducing ranking accuracy. Similarly, the knowledge graph supplies relationship information between doctors and medical entities for doctor ranking, allowing better incorporation of doctor features. This confirms that the medical knowledge graph not only enhances response quality but also plays a vital supporting role in candidate answer and doctor ranking tasks.

Removing doctor features impaired the generation task’s accuracy, diversity, knowledge utilization, fluency, patient safety, and health outcomes. Doctor features encompass not only professional background information but also capture interaction patterns between doctors and patients as well as individual treatment preferences. These elements are essential for accurately representing candidate answers.

Moreover, removing doctor features further diminishes the generation task’s ability to utilize candidate answers. In the complete model, doctor features help filter candidate answers that align with physician preferences, yielding responses better suited to clinical needs. Without this capability, the model generates less professional, personalized, and practical responses. For instance, it may fail to recognize a doctor’s preference for specific treatment plans, producing clinically inappropriate answers. This deficiency reduces response accuracy and knowledge utilization while limiting diversity, as the model defaults to generating more generic and conservative replies.

The ablation study conclusively demonstrates that MT-KGAG’s performance hinges on its biomimetic design—each component replicating a facet of clinician cognition. Just as neurosurgical expertise requires years of integrated knowledge, mentorship, and case exposure, the model’s multi-task, knowledge-aware architecture enables it to “learn” clinical perception systematically, offering a scalable template for human-aligned medical AI.

### 4.3 Case study

To intuitively demonstrate how MT-KGAG works, this chapter presents a case study. A sample was randomly selected from the test dataset, where MT-KGAG was used to generate an answer, select the most appropriate candidate answer, and recommend a suitable doctor. The candidate answers and candidate doctors were retrieved using the model’s retrieve candidate answer component, as introduced in Section 2.2. The sample includes the patient’s question, four candidate answers, four candidate doctors’ descriptions, and the ground truth answer. Table 4 presents the full sample details and the responses generated by various models. Words copied from the candidate answers are highlighted in blue, while the true labels (i.e., Candidate Answer 1 and Candidate Doctor 1) are highlighted in yellow, indicating the highest-ranked answer and doctor.

**TABLE 4:** Each model’s response to patient questions.

Item	Content
Question	My mother has suffered a cerebral hemorrhage. The right side of her body is paralyzed, and her speech is unclear. She has been hospitalized for 3 days with little improvement. What should I do? On October 26, 2011, she became immobile, began foaming at the mouth, and lost the ability to speak. Approximately 20 ml of blood was discharged. She is currently being treated at Chaoyang Hospital in Liaoning Province.
Candidate answer 1	Case Analysis: Hello, based on your description, a cerebral hemorrhage requires prompt and effective treatment. Delays in hospital intervention may lead to long-term complications. Recommendation: It is strongly recommended that the patient seek care at a certified secondary or tertiary hospital for proper treatment. The priority should be to stop the bleeding promptly. If bleeding occurs, medications that promote blood circulation and help absorb the hematoma may be considered. However, it is best to consult with the attending physicians at the hospital where the patient is receiving treatment. Please note that this is an online consultation platform—professional advice from the treating hospital is essential.
Candidate answer 2	Case Analysis: Hello, this condition carries a high risk of long-term complications, such as hemiplegia (paralysis on one side of the body) and speech difficulties. Recommendation: If feasible, consider seeking treatment in a top-tier hospital located in major cities such as Beijing or Shanghai. Notable institutions include Beijing Tiantan Hospital and Huashan Hospital in Shanghai, both of which are among the leading neurology centers in China.
Candidate answer 3	Case Analysis: Hello, this may be a sequela of cerebral hemorrhage. Unfortunately, full recovery is often difficult. A follow-up cranial CT scan is recommended to assess the current condition. Recommendation: If the scan shows no major issues, consider initiating long-term rehabilitation exercises. In addition, medications that promote blood circulation and reduce blood stasis may be beneficial. It is also important to actively manage blood pressure and blood sugar levels, and to avoid greasy or high-fat foods. Visiting the rehabilitation department of a reputable local public hospital is advised to develop a personalized recovery plan. In most cases, noticeable improvement may take 6 months to a year, though full recovery is rarely achieved.
Candidate answer 4	Case Analysis: Hello, based on the information you’ve provided, it is strongly recommended that the patient be admitted to the neurology department of a hospital for inpatient treatment. Recommendation: After the symptoms of cerebral hemorrhage are brought under control, the patient should begin rehabilitation therapy under the guidance of a hospital’s rehabilitation department. With consistent and structured treatment, recovery is possible. In daily life, it is important to maintain a balanced diet, avoid overly salty foods, engage in appropriate physical activity, and closely manage both body weight and blood pressure.
Candidate doctor 1	attention treatment cooperate rest surgery eat food recheck fatigue take infection medicine appropriate timely examination conservative sequela cerebral hemorrhage exercise physical therapy acupuncture spicy irritating rehabilitation observation control avoid absorb have a check prevention disinfection greasy relatively severe recuperation drug treatment normal nerve wound mood bleeding massage relieve catch cold blood pressure activity bend over compression drink more water persist diet formal lumbar spondylosis maintain not good hygiene promote blood circulation and remove stasis vegetables serious hot compress hospitalization improvement eat some confirm nutrition injury keep warm exclude attending doctor
Candidate doctor 2	oral administration treatment relieve surgery capsule external use eat injury every day 1 month normal handle apply irritation warm water related this is food fatigue try to rest pain cephalosporin fusidic acid ointment obvious no need two examination not severe convenient MRI exercise prolonged sitting disinfection monthly change especially fixation 1 week rotator cuff 2 weeks jumping lumbar disc bending local bed rest defecation living habits squat 2 min habit prevention constipation chili alcohol soak spicy worried infection buy running six times five compression long-term activity hit stay up late foreskin self-healing get up early squat toilet regularly develop vegetables fruits banana
Candidate doctor 3	medication treatment examination eat cooperate avoid regular but prostatitis abnormal regulate sex hormone secretion surgery food femoral head necrosis is because and so on factors exist attention local public hospital improve kidney qi deficiency difference breast enhancement stimulation testicles chromosomes related considered this way diet difficulty defecating complete recovery reexamination long-term rehabilitation exercise promote blood circulation and remove blood stasis greasy half a year joint cavity help completely only method blood supply erection check out jin’gui’shen’qi’wan medication breast size menstrual cycle theory kidney yang deficiency six items urology hormone normal endometrial
Candidate Doctor 4	helpful analysis treatment hot water drink quit drinking quit smoking avoid eating cold spicy medication eat some regular necessary diet cold exercise eat attention symptomatic treatment like prevention appropriate time to clarify diagnosis gastritis reasonable rest less personal hygiene local don’t eat more control topical provide weight blood pressure too full sweet gastroenterology combine anti-inflammatory drugs hospitalization enhance physique regulate physical condition blood lipids blood sugar omeprazole vegetables fruits increase nutrition drink some vitamin B traditional Chinese medicine TCM Chinese herbal medicine effective rule out domperidone surgery hygiene food epilepsy child timely add clothing
Ground truth	Hello, based on what you described, cerebral hemorrhage is confirmed. Generally, the peak period of cerebral edema occurs 3–5 days after the hemorrhage, which is relatively dangerous, and the condition may fluctuate within 2 weeks. At present, the main goal is to stabilize the condition. Once the condition is stable, rehabilitation treatment should be initiated. There are usually some sequelae, but with good rehabilitation, some patients can regain independence in daily life. Recommendations: 1. Bed rest and maintaining smooth bowel movements (straining during defecation or getting out of bed can increase the risk of re-bleeding). 2. Stabilize blood pressure. 3. Medication treatment.
MT-KGAG	Top-ranked candidate answer: candidate answer 1; Top-ranked candidate doctor: candidate doctor 1; Generated response: I’m sorry to hear that your mother has suffered a cerebral hemorrhage. I hope she recovers soon. Here are some suggestions and information: Consult the attending hospital: Follow the doctor’s advice and adhere to the treatment plan, including medication, diet, and rehabilitation exercises. Try to maintain your mother’s physical stability to support better recovery. Dietary adjustment: According to the doctor’s recommendations, ensure your mother’s diet is healthy and balanced. Moderately increase protein and nutrient-rich foods to aid the healing process. Adequate rest: Make sure your mother gets enough rest and sleep, which is crucial for the recovery of brain function. As for whether cerebral hemorrhage will shorten life expectancy or whether your mother can walk or live like a normal person again, it depends on the specific case and the effectiveness of the treatment. If not treated in time, it can result in sequelae. However, with proper treatment, it is possible to help your mother regain functionality in daily life and improve quality of life. Everyone’s condition is different, so it is important to carry out a personalized assessment and adjustments under the doctor’s guidance.
w/o multi-task learning	Generated response: Patients with cerebral hemorrhage need to seek medical attention promptly. A CT scan is recommended, and treatment should follow the doctor’s advice. Rehabilitation training can aid in recovery, but the effectiveness varies from person to person.
w/o medical knowledge graph	Generated response: An EEG (electroencephalogram) may be needed. If the issue is vascular and the cerebral hemorrhage has affected a certain functional area of the brain, corresponding symptoms can appear. If the patient only has a headache, it might not be serious. If there are no other problems, medication may not be necessary. However, if symptoms persist, doctors generally recommend oral medication and active diagnosis.
w/o doctor feature	Top-ranked candidate answer: candidate answer 3; Generated response: The treatment of cerebral hemorrhage requires timely hemostasis and reduction of intracranial pressure, and hospitalization is recommended. During the recovery period, physical therapy and speech training can help improve motor and language functions. The prognosis of cerebral hemorrhage varies from person to person—some patients may regain the ability to walk, but long-term rehabilitation training is often necessary.

MT-KGAG leverages a multi-task learning framework to collaboratively perform medical Q&A through three core modules. First, multi-task learning includes ranking candidate answers and doctors, enabling the model to match questions with the most appropriate doctors and their expert responses. Second, the model incorporates external medical knowledge via a neurosurgical knowledge graph, enhancing its reasoning capabilities and medical specificity. Finally, by introducing doctor features, the model strengthens the representation of both the question and the candidate answers. In this case, MT-KGAG successfully identified Candidate Doctor 1 as the most suitable responder and ranked Candidate Answer 1 as the best answer, supported by joint training. During answer generation, the model utilized the knowledge graph for medical reasoning and accurately copied domain-specific terms from the candidate answers, resulting in high-quality medical advice. The joint training of sub-tasks improved each component’s performance, producing responses that are both professional and highly targeted.

When multi-task learning was removed, the model could no longer optimize candidate doctor and answer ranking simultaneously, leading to overall performance degradation. Specifically, without shared representations and joint optimization, the model failed to effectively link doctor features with answer relevance. As a result, the generated answers became more generic and less actionable. For instance, in a cerebral hemorrhage case, the model without multi-task learning might generate vague suggestions like “seek medical attention promptly” instead of providing concrete treatment or rehabilitation guidance, reducing its ability to handle complex medical scenarios.

Removing the medical knowledge graph had little impact on ranking performance, as the model could still correctly rank Candidate Answer 1 and Candidate Doctor 1, suggesting that the ranking tasks rely primarily on semantic matching between the question and candidate texts. However, answer generation quality dropped significantly. Without the knowledge graph, the model lacked the ability to perform informed reasoning and could only copy words from the candidate answers mechanically. The knowledge graph offers rich background knowledge that supports professional reasoning during response generation. Without it, the model struggles to explain conditions, treatment plans, or recovery advice accurately, reducing the depth and professionalism of its responses.

Lastly, when doctor features were removed, performance declined in both candidate answer ranking and answer generation. Specifically, Candidate Answer 3 was incorrectly ranked highest instead of the correct Candidate Answer 1, indicating the critical role of doctor features in ranking tasks. These features enhance the model’s ability to represent interactions between patients and doctors. Without them, the model fails to accurately assess the relevance of candidate answers, leading to incorrect rankings. Additionally, the generated answers lacked personalization and practical value due to the absence of doctor-specific context.

## 5 Conclusion

This study proposes the MT-KGAG model, a perception-aware intelligent Q&A framework for neurosurgery, designed to emulate clinician cognitive workflows through two innovations: (1) perception-anchored knowledge fusion, integrating neurosurgical knowledge graphs and doctor expertise profiles to ground responses in structured medical ontologies and real-world clinical reasoning; (2) cognitive-aligned multi-task synergy, jointly optimizing answer generation, ranking, and recommendation tasks to mirror the integrative decision-making patterns of physicians. Through comparative analysis and ablation studies, MT-KGAG significantly improves the performance of the automated question-answering system across multiple dimensions and outperforms baseline models in ranking tasks for candidate answers and physician selection.

At the theoretical level, this study provides profound insights into the development of intelligent medical question-answering systems in neurosurgery. The MT-KGAG model advances the application research on combining retrieval models with generative models, further enriching the technical framework in this field. Specifically, MT-KGAG first retrieves candidate answers for questions using a retrieval model, then encodes the question, candidate answers, the physicians providing those answers, and the medical knowledge graph simultaneously in the encoder. Finally, these encoded representations are fused and input into the decoder. This design enables the model to fully leverage information from diverse data sources, thereby enhancing its performance in neurosurgery. This bridges the gap between retrieval systems’ rigidity and LLMs’ hallucination risks, advancing hybrid AI toward human-like diagnostic reasoning.

At the practical level, MT-KGAG demonstrates unique advantages and flexibility. First, by embedding physician characteristics in the physician recommendation task, the system cannot only provide accurate diagnostic suggestions but also recommend relevant experts based on patient needs, forming a dual-service model of “AI advice + physician matching.” This innovative service model offers users more personalized and comprehensive medical support. Second, while maintaining high accuracy, MT-KGAG adopts a modular decoupling design, meaning that subtasks (e.g., retrieval, generation, recommendation) can be flexibly configured for different application scenarios, adapting to the digital infrastructure of various medical institutions. This design ensures strong generalizability and adaptability, meeting the needs of diverse medical environments.

However, this study also has some limitations. First, the current candidate answer retrieval employs only basic matching strategies and does not fully account for dynamic user feedback. In the future, a reinforcement learning-based dynamic retrieval mechanism will be a key direction for improving system performance, enhancing answer relevance and accuracy through real-time retrieval strategy adjustments. Second, with technological advancements, future research could explore migrating the MT-KGAG framework to larger-scale pre-trained language models (e.g., DeepSeek, GPT-4) and further optimizing its application in the medical field through fine-tuning techniques. The advantage of large language models lies in their powerful contextual understanding and language generation capabilities, which could provide higher-level intelligent support for medical question-answering systems.

## Data Availability

The original contributions presented in this study are included in this article/supplementary material, further inquiries can be directed to the corresponding author.

## Footnotes

1
http://www.xywy.com/
